# Effect of *Purgative Manna* on Neonatal Hyperbilirubinemia: A Systematic Review and Meta-analysis

**DOI:** 10.22037/ijpr.2019.2388

**Published:** 2019

**Authors:** Firoozeh Sajedi, Shiva Fatollahierad

**Affiliations:** *Pediatric Neurorehabilitation Research Center, University of Social Welfare and Rehabilitation Sciences, Tehran, Iran. *

**Keywords:** Purgative manna, Neonate, Hyperbilirubinemia, Management, Meta-analysis

## Abstract

The aim of this systematic review was to evaluate the effect of *purgative manna *on the unconjugated hyperbilirubinemia in neonates. Pubmed, Scopus, Chochrane library, Iranmedex and Google scholar were last searched in February 2017. Randomized controlled trials that evaluated the effect of *purgative manna* on the treatment of neonatal hyperbilirubinemia were included in the review. For meta-analysis, weighted mean difference (WMD) with 95% confidence interval (CI) was used. The outcomes of interests were serum bilirubin levels and length of hospital stay in neonates with jaundice. Seven randomized controlled trials with 812 neonates were eligible to be included in this systematic review. The meta-analysis included six of seven controlled trials. Bilirubin levels were significantly lower at 12 h (WMD: -1.48, 95% CI: -2.31 to -0.65), 24 h (WMD: -2.47, 95% CI: -3.22 to -1.71), 36 h (WMD: -2.83, 95% CI: -4.87 to -0.80), 48 h (WMD: -1.49, 95% CI: -2.36 to -0.63) and 72 h (WMD: -0.68, 95% CI: -1.28 to -0.08) following intervention in *purgative manna *group. Length of hospital stay was also decreased in *purgative manna *group (WMD: -0.93, 95% CI: -1.35 to -0.50). Finally, *purgative manna *administration decreased serum bilirubin level and length of hospital stay in neonates with unconjugated hyperbilirubinemia. More studies are needed to evaluate the efficacy, dosage, and side effects of *purgative manna*.

## Introduction

Jaundice is one of the most common problems in neonates (1, 2). The prevalence of severe hyperbilirubinemia was 12 percent in the north of Iran in 2005 and 15 percent in the south of Iran in 2010 (3, 4). Neonatal hyperbilirubinemia is mostly benign but high serum bilirubin levels may cause kernicterus, brain damage, and consequently developmental delay (5-6).

Phototherapy is the conventional treatment method for neonatal hyperbilirubinemia in recent years; however, many infants need exchange transfusion (ET) if phototherapy could not reduce the total serum bilirubin level in them effectively. ET could cause morbidities such as necrotizing enterocolitis, acute renal failure, and even death in neonates (7). In addition, phototherapy itself might have side effects and can be harmful especially in extremely low-birth-weight (ELBW) newborns (8). Retinal degeneration, diarrhea, dehydration, skin rash (9), oxidative stress-related diseases such as necrotizing enterocolitis and patent ductus arteriosus are reported adverse effects of phototherapy (10). Allergic diseases such as asthma and allergic rhinitis (11), nevi, apoptosis and DNA damage in peripheral blood lymphocytes of full-term infants (1) have been found to be higher with this treatment method. 

As a result of these side effects, other treatment methods are studied to decrease the duration of phototherapy and reduce the chance of ET in neonates. Complementary medicine such as *purgative manna *administration in Iran was used for the treatment of neonatal jaundice before phototherapy became common practice (12). 

*Purgative manna *is a white rather yellow sweet substance produced by an insect on Cotoneaster genus of Rosaceae Family which is called *shirkhesht* in Persian (13). The most substances in *purgative manna *are mannitol, fructose, glucose, and sucrose. About 40 to 60 percent of *manna *is mannitol (14).

The laxative effect of *manna* might interrupt bilirubin enterohepatic circulation and reduce indirect bilirubin. Several studies evaluated the effect of *purgative manna *on neonatal jaundice. They reported that *manna* could decrease the duration of phototherapy by reducing bilirubin levels in icterus neonates (15-17). Thus, we carried out this systematic review and meta-analysis to assess the effect of *purgative manna *in combination with phototherapy on the management of unconjugated hyperbilirubinemia in neonates.

## Experimental

*Methods*

We searched Pubmed, Medline, Scopus, Cochrane library, Iranmedex, and Google scholar in February 2017 to identify all relevant and published studies about the effect of *purgative manna *on neonatal jaundice. The database searches were performed using the medical subject heading terms “*purgative manna*”, “hyperbilirubinemia”,“hyperbilirubinemia, neonatal”, “jaundice”, “jaundice, neonates” and key words “*purgative manna*”, “*shirkhesht*”, “*shir khesht*”, “bilineaster”, “*cotoneaster*”, “jaundice”, “hyperbilirubinemia”, “bilirubin”.

The reference lists of identified studies and review articles were also hand searched. The search of databases was not restricted by language. 

The titles and abstracts of the relevant studies were assessed for eligibility. The studies appearing eligible based on their abstracts were read in full. 

The studies were included if they were randomized controlled trials (RCT) and assessed the effect of *purgative manna *as a treatment method on unconjugated hyperbilirubinemia in neonates. Articles that evaluated the effect of *purgative manna *on prevention of neonatal hyperbilirubinemia or examined the effect of *purgative manna in-vitro* were excluded.

The following data were extracted from included studies by two of review authors: author, publication year, number of participants, characteristics of neonates (gestational age, birth weight, nutrition, postnatal age and total serum bilirubin level on hospital admission), dose of *purgative* *manna*, *purgative manna *sources, side effects of treatment and outcomes. The unstated details were obtained through communication with the authors of included studies.

Furthermore, the risk of bias of studies was evaluated independently by the two reviewers using “the Cochrane Collaborations’ tool for assessing risk of bias” (23). Therefore, the methods of randomization, allocation concealment, blinding of participants and personnel, blinding of outcome assessment, incomplete outcome data, and selective reporting were assessed for each study.

Each of the mentioned domain was assessed as ‘low risk of bias’, ‘high risk of bias’, or ‘unclear risk of bias’. Disagreements between the reviewers about the risk of bias for any domain were resolved by consensus.

The outcomes of interest were serum bilirubin levels at 12, 24, 36, 48, and 72 h after the start of intervention, the length of hospital stay, and the frequency of defecation per day in neonates. 

We used Review Manager 5.3 (The Cochrane Collaboration) to do the meta-analysis and write the review. 

The pooled effects of *purgative manna *on bilirubin levels were calculated as weighted mean difference (WMD) with 95% CI. WMD below zero suggests benefit to the intervention groups (if confidence interval does not include the zero line) because bilirubin is expected to decrease with time. Fixed effects or random effects model was used for the meta-analysis according to the test of heterogeneity (I^2^). If I^2 ^was less than 50, then the fixed effects model was employed. A *p*-value < 0.05 was considered statistically significant.

## Results

**Figure 1:**
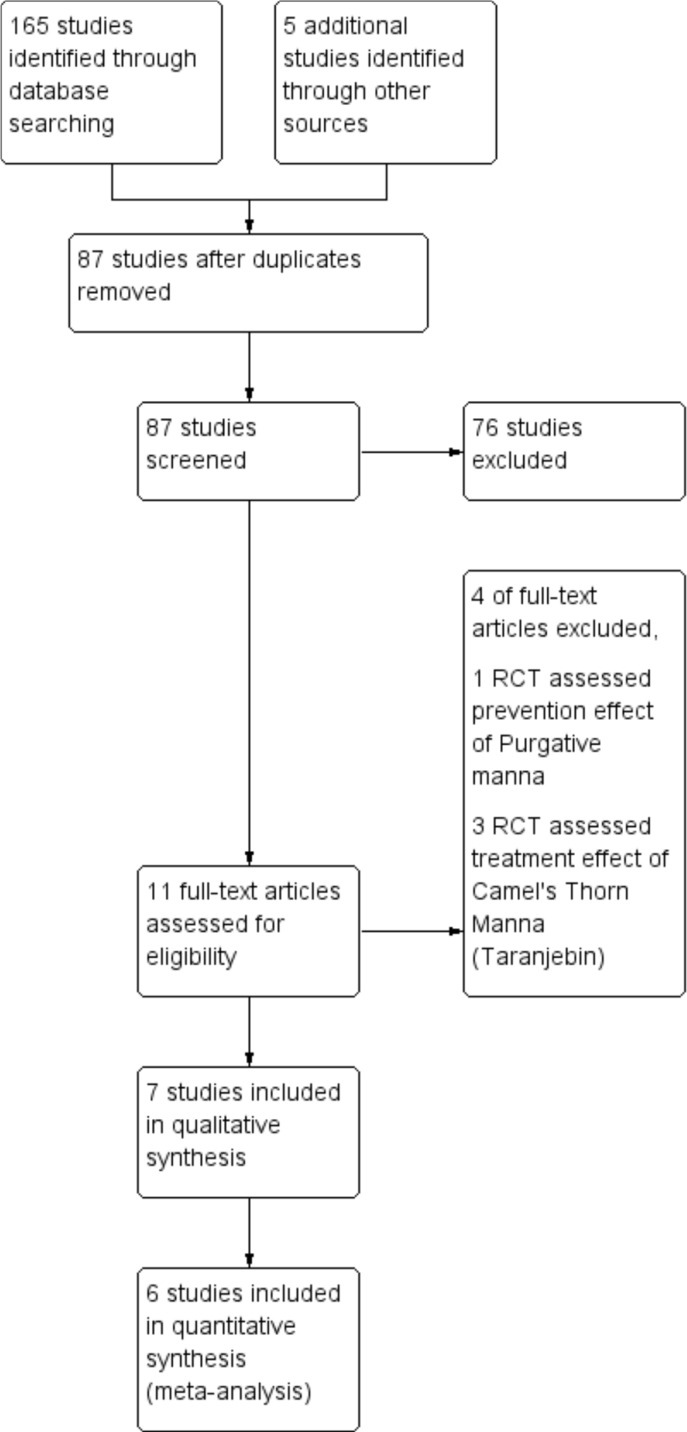
Flow diagram of study

A total of 165 articles from different databases were identified. After reviewing the full text of articles, only seven clinical trials with 812 neonates met the inclusion criteria and were included in this review (Figure 1). The languages of selected studies were in Persian (16, 17 and 19) and English (18, 20-22).

**Figure 2:**
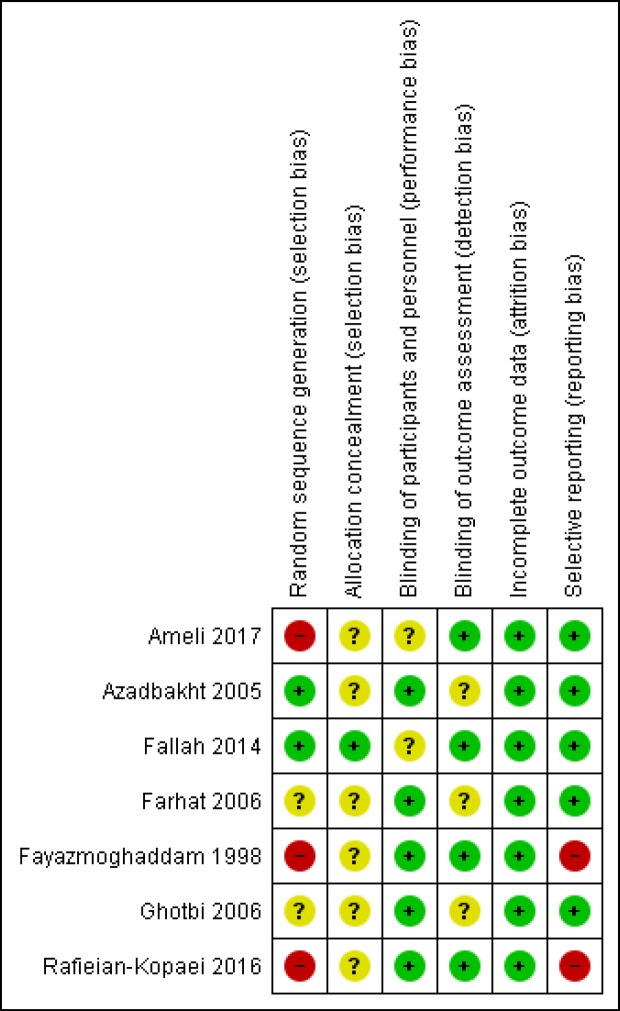
Risk of bias summary (Reviewers’ assessment of each risk of bias item; “+”, low risk of bias; “?”, unclear risk of bias; and “−”, high risk of bias)

**Figure 3:**
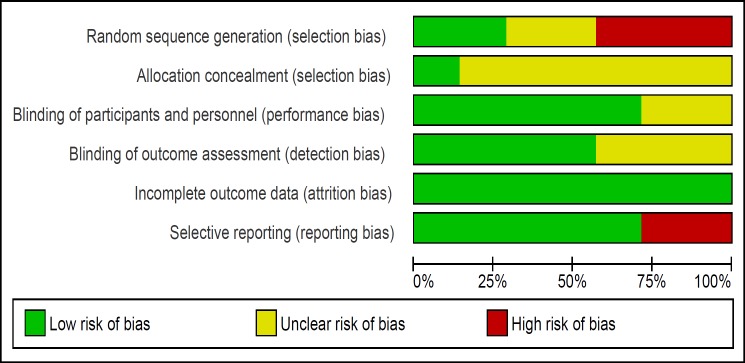
Risk of bias graph (Review authors' judgments about each risk of bias item presented as percentages across all included studies)

**Figure 4:**
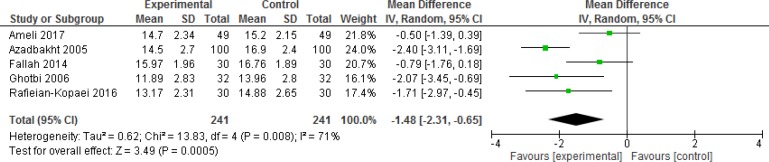
Comparison of bilirubin level at 12 h

**Figure 5:**
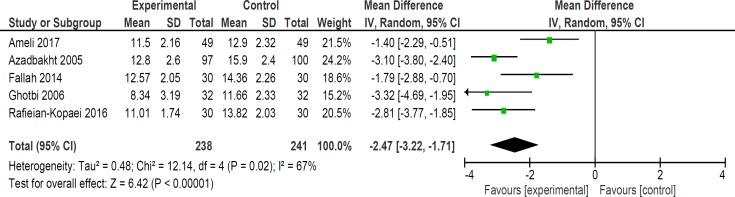
Comparison of bilirubin level at 24 h

**Figure 6:**
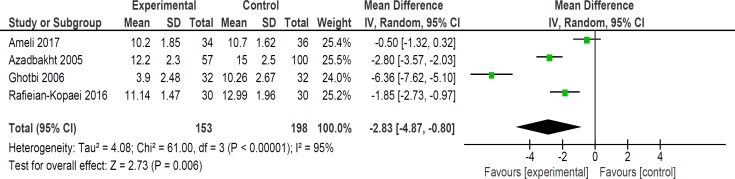
Comparison of bilirubin level at 36 h

**Figure 7:**
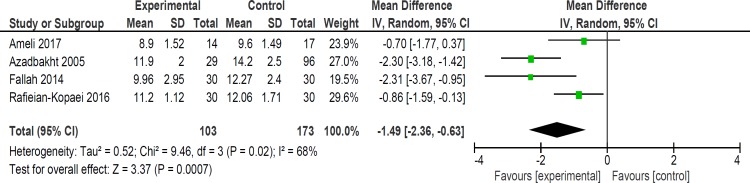
Comparison of bilirubin level at 48 h

**Figure 8:**
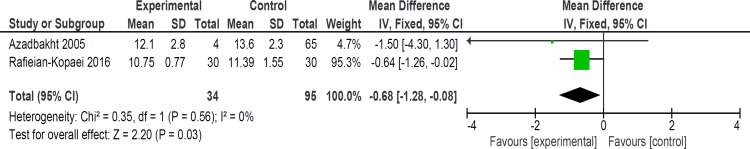
Comparison of bilirubin level at 72 h

**Figure 9:**
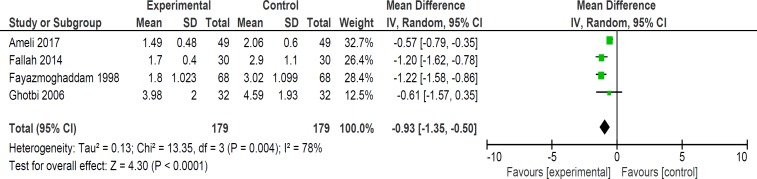
Comparison of length of hospital stay in days

**Table 1:** Characteristics of included studies

**Author, year (reference)**	**Date and Location**	**Participants**	**TBS level on admission**	**Intervention**	***Purgative manna*** **dose (mg)**	***Purgative manna *** **source**
Fayazmoghaddam, 1999 (16)	October 1998, Sanandaj, Iran	136 neonates	Admission on 24 h to 72 h after birth: 15-20 mg/dL Admission on more than 72 h after birth: ≥20 mg/dL	Intervention group (n = 68): *Purgative manna *three times a day + phototherapy Control group (n = 68): water (placebo) three times a day + phototherapy	666.67 mg *purgative manna *three times a day	Not reported
Azadbakht, 2005 (17)	Iran	200 neonates Full-term	Unclear	Intervention group (n = 100): 5 drop *purgative manna *three times a day + phototherapy Control group (n = 100): placebo drop three times a day + phototherapy	152.5 mg mannitol* three times a day	*Manna *of Cotoneaster discolor pojark from Qaien, Khorasan, Iran
Farhat, 2006 (18)	May 2001 to December 2003 Mashhad, Iran	104 neonates Breastfed, more than 2500 g at birth	18-29 mg/dl	Intervention group (n = 50): a single dose of 6 g of *purgative manna *dissolved into distilled water (8 mL) during the first hour of trial + phototherapy Control group (n = 54): a single dose of starch solution (0.1%, 8 mL) colored with one drop of caramel solution (placebo) + phototherapy	6000 mg *purgative manna *for one dose	*Manna *of cotoneaster tricolor pojark from Qaien, Khorasan, Iran
Ghotbi, 2006 (19)	June 2003 to February 2006, Iran	64 neonates Full- term, Breastfed, 2500-4000 g at birth,beginning of jaundice: 3-11 days after birth	15-20 mg/dL	Intervention group (n = 32): 10 cc *purgative manna *three times a day for the duration of one day + phototherapy control group (n = 32): distilled water (placebo) three times a day + phototherapy	1666.67 mg *purgative manna* three times	*Manna *discolor from Khorasan
**Author, year (reference)**	**Date and Location**	**Participants**	**TBS level on admission**	**Intervention**	***Purgative manna*** **dose(mg)**	***Purgative manna *** **source**
Fallah, 2014 (20)	September 2012 to February 2013 Yazd, Iran	90 neonates Full-term, breastfed, 2500-4000 g at birth, beginning of jaundice: 3-7 days after birth	15-20 mg/dL	Intervention groups (n = 60): Thirty neonates were given 5 drop per kg Bilineaster drop every 8 h up to 48 h + phototherapy Thirty neonates received half of glycerin suppository every 12 h up to 48 h + phototherapy Control group (n = 30): phototherapy	75 mg/kg Mannitol* three times a day	Bilineaster drop from Sobhan Darou Company
Rafieian-Kopaei, 2016 (21)	2010 Shahrekord, Iran	120 neonates Full-term, breastfed, 2500-4000 g at birth, beginning of jaundice: 2 days after birth	14-20 mg/dL	Intervention groups (n = 90): Thirty neonates were given 3 drop per kg Bilineaster drop three times a day + phototherapy and their mothers were given dose of neonates × 3 Bilineaster drop Thirty mother were given dose of neonates × 3 Blineaster drop Thirty neonates were given 3 drop per kg Bilineaster drop three times a day + phototherapy Control group (n = 30): distilled water (placebo) three times a day + phototherapy	Unidentified	Bilineaster drop from Barij Essence Pharmaceutical Company
Ameli, 2017 (22)	June 2015 to March 2016 Mashhad, Iran	98 neonates Term (35-42 weeks), breastfed, more than 2000 g at birth, beginning of jaundice: 2-14 days after birth	More than 17-20 mg/dL	Intervention group (n = 49): 5 drop per kg Bilineaster drop three times a day + phototherapy Control group (n = 49): phototherapy	75 mg/kg Mannitol* three times a day	Bilineaster drop from Sobhan Darou Company

* The main constituent of *manna *is mannitol of which it may contain from 40 to 60 percent.

**Table 2:** Results of included studies

**Author, year (reference)**	**Outcomes**	**Results**	**Side effects in intervention group**
Fayazmoghaddam, 1999 (16)	Length of hospital stay	Length of hospital stay were lower in intervention group (*p *≤ 0.001)	Not reported
Azadbakht, 2005 (17)	Bilirubin level, Length of hospital stay	Bilirubin levels were lower in intervention group at 12, 24,36 and 72 h after treatment (*p *≤ 0.05) Discharge of the intervention group from hospital started after 1.5 days of hospitalization. For the control group, the beginning of release from hospital was after 2.5 days of hospitalization.	Small number of neonates developed listlessness caused by loss of water (as a result of osmotic diarrhea due to *purgative manna *or phototherapy)
Farhat, 2006 (18)	Bilirubin level, Defecation frequency	Bilirubin levels and defecation frequency of intervention group were not significantly different from control group	No side effects (follow up at 24 h after phototherapy discontinued)
Ghotbi, 2006 (19)	Bilirubin level, Length of hospital stay, Defecation frequency	Bilirubin levels were lower in intervention group at12, 24 and 36 h after treatment (*p *≤ 0.001) Length of hospital stay was lower in intervention group (*p *≤ 0.001) Defecation frequency was significantly higher in intervention group (*p *≤ 0.001)	Small number of neonates developed listlessness caused by loss of water (as a result of osmotic diarrhea due to *purgative manna *or phototherapy)
Fallah, 2014 (20)	Bilirubin level, Length of hospital stay, Defecation frequency	Bilirubin levels were lower at 24 h (*p *= 0.02) and 48 h (*p *= 0.02) after treatment in *purgative manna *group Length of hospital stay was lower in *purgative manna *group versus control group (*p *= 0.02) Defecation frequency was not significantly different between *purgative manna *group and control group	No side effects (during hospitalization and on the second day after discharge in the clinic of hospital, no side effects were seen in *purgative manna *group)
Rafieian-Kopaei, 2016 (21)	Bilirubin level, Length of hospital stay	Bilirubin levels were lower in intervention group at 12, 24 and 36 h after treatment (*p *≤ 0.05) Length of hospital stay was lower in intervention groups versus control group (*p *≤ 0.001)	No side effects
Ameli, 2017 (22)	Bilirubin level, Length of hospital stay	Bilirubin levels were lower at 24 h (*p *< 0.001) after treatment in *purgative manna *group Length of hospital stay was lower in *purgative manna *group versus control group (*p *< 0.001)	No side effects (follow up one and two months after discharge by phone calls)

The risk of bias of studies was evaluated by two authors. All seven trials were RCTs however three of them had used alternation as the method of randomization (16, 21- 22) and only one study described the method of allocation concealment (20). The risk of bias summary and graph of included studies are summarized in Figures 2 and 3.

The dates of trials were from 1999 to 2017. Five of trials included only term infants (17, 19-22) and two of them included both term and preterm neonates (16, 18). 

Neonates in six trials received *purgative manna *orally every eight hours. One of these six trials administered *manna* for only one day (19) and one trial administered *manna* for 48 h (20). In one study neonates just received *manna* for a single oral dose during the first hour of hospital admission (18). In addition, all neonates in the control and intervention groups of seven included trials received phototherapy on hospital admission and during hospitalization. The characteristics of included studies are presented in Table 1.

The preparation methods of *purgative manna *solution for neonates in included studies were as follows:

Fayazmoghaddam *et al.* dissolved one gram of *purgative manna *into 30 mL water for 10 neonates every day (33.33 mg purgative *manna*/mL). Afterwards, 20 cc of solution were administered to every neonate three times a day (16).

Azadbakht *et al.* obtained *manna* from Khorasan, a province of north eastern Iran. They dissolved 400 g of *manna* into 400 mL of distilled water and dried it under reduced pressure. Propyl paraben and methyl paraben were used as preservative. Finally, the dry extract had 88.3% *manna* from which 50-60% was mannitol. One mL of the drop contained 610 mg active constituents based on mannitol (610 mg mannitol/mL). The neonates in intervention group received five drops of *purgative manna *solution three times a day (17).

Farhat *et al.* collected *purgative manna *from Khorasan. They dissolved six grams of *manna* into distilled water (8 mL) for every neonate (750 mg *purgative* *manna*/mL) (18).

Ghotbi *et al.* gathered *purgative manna *from Khorasan. They divided *manna* to five gram packages. Every package was for administration to one neonate for one day. Five grams of *manna* was dissolved into 30 mL boiling water every day (166.67 mg *purgative* *manna*/mL). Every neonate in intervention group received 10 mL of prepared solution three times a day for the duration of one day (19).

Fallah *et al.* (20) and Ameli *et al.* (22) used Bilineaster drop from Sobhan Darou Company (300 mg mannitol/mL) and Rafieian-Kopaei *et al.* (21) used Bilineaster drop from Barij Essence Pharmaceutical Company.

In three trials, the number of neonates who needed exchange transfusion or Phenobarbital were reported. Azadbakht *et al.* reported that three neonates in control group had exchange transfusion. In addition, Phenobarbital or *purgative manna *was administered to four neonates in control group to manage jaundice. None of the neonates in intervention group needed exchange transfusion or Phenobarbital (17). Furthermore, in Fallah *et al.* study one neonate in each group of trial required exchange transfusion (20). Ameli *et al.* also reported that one neonate in *purgative manna *group and five neonates in control group received Phenobarbital and eleven neonates in control group underwent phototherapy again after discharge for the management of their hyperbilirubinemia (22). The obtained results of trials are summarized in Table 2.

*Meta-analysis findings*

In meta- analysis, one study was not included because they administered *purgative manna *only for one dose (18) and the rest of studies administered *manna* three times a day. Thus, the intervention was very different from the rest of studies. Three trials had more than one intervention group. As a result, among 708 neonates in included studies of meta-analysis, only 618 neonates were considered for analysis and 90 neonates in intervention groups other than *purgative manna *were excluded.

Mean bilirubin level collected from neonates at 12 h after the start of intervention was lower for those allocated to *purgative manna *and phototherapy than placebo and phototherapy (5 trials, 482 neonates) (WMD: -1.48, 95% CI: -2.31 into -0.65). Since WMD show reduction of bilirubin with 1.48 and CI does not contain zero, the intervention is statistically significant (Figure 4). Similarly, five trials evaluating 479 neonates found significantly lower levels of neonatal bilirubin in *purgative manna *group at 24 h after the start of intervention (WMD: -2.47, 95% CI: -3.22 to -1.71) (Figure 5). At 36 h following intervention, four trials showed *purgative manna *group had lower level of bilirubin versus control one (351 neonates) (WMD: -2.83, 95% CI: -4.87 to -0.80) (Figure 6). In addition, four trials assessing neonatal bilirubin level at 48 h (276 neonates) (WMD: -1.49, 95% CI: -2.36 to -0.63) (Figure 7) and at 72 h after the onset of intervention, (2 trials, 129 neonates) (WMD: -0.68, 95% CI: -1.28 to -0.08) showed lower levels of bilirubin in *purgative* *manna* group (Figure 8).

Although six trials assessed the length of hospital stay in neonates but two of studies’ data were not available for quantitative analysis. In four studies, length of hospital stay was lower in *purgative manna *group versus control group (358 neonates) (WMD: -0.93, 95% CI: -1.35 to -0.50) (Figure 9) (16, 19, 20 and 22).

Defecation frequency was another outcome that was considered in two trials. Ghotbi *et al.* reported increasing number of defecation per day in intervention group (one trial) (64 neonates) (WMD: 0.71, 95% CI: 0.29 to 1.13) (19). However, Fallah *et al.* showed that number of defecation per day at 24 h (one trial) (60 neonates) (WMD: 0.65, 95% CI: -0.19 to 1.49) and 48 h (one trial) (60 neonates) (WMD: 1, 95% CI: 0.1 to 1.90) following intervention were not significantly different in both groups (20).

## Discussion

We found that *purgative manna *administration reduced bilirubin levels at 12, 24, 36, 48, and 72 hours following treatment. In addition, our review revealed that *purgative manna *decreased the length of hospital stay and the duration of phototherapy. No significant side effects were seen in neonates during hospitalization and follow ups. The effect size of bilirubin level was more notable at 36 h (WMD: -2.83) compared with 12 and 24 h (WMD: -1.48 and -2.47 respectively). However, the effect size decreased at 48 h (WMD: -1.49) and 72 h (WMD: -0.68). This conclusion could be the result of lesser effect of treatment on lower blood bilirubin levels at 2 to 3 days after initiation of therapy and small number of still admitted neonates. 

The majority of studies were incorporated just term infants without any hemolytic disorders. However, preterm infants are at increased risk of long exposure to phototherapy and its side effects. In addition, only in Fayazmoghaddam *et al.* and Farhat *et al.* studies, infants with a bilirubin level of more than 20 mg/dL were included (16, 18) and in the rest of studies the range of bilirubin level was 14 to 20 mg/dL on admission (19-22). Thus, more studies are needed to evaluate the efficacy of *manna* on preterm neonates and higher serum bilirubin levels in infants with jaundice.

Although the administration frequency of *purgative manna *was every eight hours in six studies, *manna* sources were not the same in them. The *manna* that was used in included studies, was from different companies or region. Thus their prepared drops might had different potency which leads to different results. Furthermore, based on the review, there were no serious side effects in neonates who received *purgative manna *during treatment but the majority of the studies didn’t evaluate infants in the following months of therapy. In respect to the importance of understanding any serious side effects of *manna*, long term follow up studies are needed. Hence we could not draw any precise conclusion about any adverse effects of *manna*.

We showed that length of hospital stay and consequently duration of phototherapy reduced significantly in intervention groups. This result is important since it can decrease any probable side effects of phototherapy. In addition, by staying less time in hospital, the cost of hospital stay and anxiety of parents will lessen.

*Purgative manna *might have laxative effect; therefore, it decreases bilirubin reabsorption by the enterohepatic circulation. However, just in one study, the defecation frequency was higher in intervention group than the control one (19) and in another study, *manna* did not have any significant effect on defecation number per day (20). Other studies did not have report this outcome.

The validity of drawing conclusions should be interpreted carefully. Small number of including participants in each outcome analysis is a weakness for this meta-analysis. Only 482 neonates at 12 h, 479 neonates at 24 h, 351 neonates at 36 h, 276 neonates at 48 h and 129 neonates at 72 h were compared between the efficacy of *purgative manna *and phototherapy treatment with phototherapy alone. In addition, six out of seven trials didn’t report the allocation concealment and just three studies were double blinded. Therefore, further well designed studies with larger sample sizes are needed to ascertain the actual efficacy of *purgative manna *for the management of neonatal hyperbilirubinemia.

In summary, oral administration of *purgative manna *could reduce the bilirubin level in neonates and in consequence the duration of phototherapy. Thus, *purgative manna *could be used in combination with phototherapy to manage unconjugated hyperbilirubinemia in neonates. However, there is a need for more well designed studies to assess the efficacy, dosage, and any probable side effects of *purgative* *manna*. 
